# Comparative preventive effects of probiotic and postbiotic preparations of *Lacticaseibacillus rhamnosus* L156.4 and GG in a 5-FU-induced mucositis model

**DOI:** 10.1080/29933935.2026.2675856

**Published:** 2026-05-21

**Authors:** Gabriela Munis Campos, Rhayane Cristina Viegas Santos, Ludmila Silva Quaresma, Monique Ferrary Américo, Eduarda Guimarães Sousa, Gabriel Camargos Gomes, Andria dos Santos Freitas, Fernanda Alvarenga Lima Barroso, Renato Elias Moreira Júnior, Ana Lucia Brunialti Godard, Renan Pedra de Souza, Enio Ferreira, Luiz Octávio Pires, Simone Odília Antunes Fernandes, Valbert Nascimento Cardoso, Marcus Vinícios Canário Viana, Milca da Silva Figueiredo Siqueira, Rodrigo Dias de Oliveira Carvalho, Luís Claúdio Lima de Jesus, Tulio Marcos Santos, Yves Le Loir, Eric Guédon, Vasco Azevedo

**Affiliations:** a Department of Genetics, Ecology, and Evolution, Federal University of Minas Gerais, Belo Horizonte, Brazil; b Department of Pathology, Federal University of Minas Gerais, Belo Horizonte, Brazil; c Faculty of Pharmacy, Federal University of Minas Gerais, Belo Horizonte, Brazil; d Institute of Biology, Federal University of Bahia, Salvador, Brazil; e Uniclon Biotecnologia, Bioinformatics Services and Probiotics, Belo Horizonte, Brazil; f STLO, INRAE, L'Institut Agro Rennes-Angers, Rennes, France

**Keywords:** Chemotherapy-induced mucositis, probiotic therapies, inflammation, microbial diversity and dysbiosis

## Abstract

Intestinal mucositis is a debilitating side effect of chemotherapy, characterized by epithelial damage, inflammation, and microbial dysbiosis, that significantly impairs patients' quality of life. This study evaluated the therapeutic potential of *Lacticaseibacillus rhamnosus* L156.4 in its live, heat-inactivated, and supernatant forms, compared to the reference strain *L. rhamnosus* GG (LGG), in a murine model of 5-fluorouracil (5-FU)-induced mucositis. Preventive treatment with live L156.4 significantly reduced mucositis-induced intestinal damage, preserving villus length, crypt depth, and goblet cell numbers, thereby supporting barrier integrity. In contrast, heat-inactivated and supernatant forms provided only limited protection. Both live L156.4 and LGG reduced myeloperoxidase activity and downregulated inflammatory markers such as *Il1b* and *Nfkb1*, confirming anti-inflammatory effects. They also modulated tight junction-related gene expression with L156.4 showing strong induction of *Muc2*, suggesting enhanced epithelial protection. Fecal microbiota analysis showed that, whereas LGG restored the loss of bacterial diversity and evenness induced by 5-FU, L156.4 primarily modulated microbiota composition by restoring the abundance of specific phyla and genera. Overall, our findings identify L156.4 as a promising probiotic candidate, with effects comparable to and complementarity with LGG, for mitigating chemotherapy-induced mucositis. Further studies are required to assess dose effects and long-term safety.

## Introduction

Intestinal mucositis is a severe chemotherapy side effect, affecting up to 90% of patients.^1^ It results from inflammation of the intestinal mucosa and is a common consequence of anticancer treatments.^2^ This condition can lead to serious complications, including dehydration, malnutrition, sepsis, and, in severe cases, death.^3^ Additionally, mucositis can significantly impact treatment adherence and quality of life, posing a significant threat to the overall health of chemotherapy patients.^4,5^ As an effect of mucositis, there is an increase in intestinal permeability and the risk of bacterial translocation, both of which contribute to the onset of sepsis.^6,7^ Furthermore, chemotherapy-induced mucositis disrupts the gut microbiota, causing dysbiosis, which worsens inflammation and exacerbates intestinal damage.^8^ This imbalance in the microbiota affects intestinal homeostasis and compromises the gut barrier, increasing the severity of mucositis and its related complications.^9^

Given these challenges, strategies to alleviate chemotherapy-induced mucositis increasingly focused on restoring microbial balance and reducing inflammation through targeted interventions, such as probiotics and other microbiota-modulating therapies.^10^ In this context, probiotics and postbiotics have gained significant attention as alternative adjuvants for reducing inflammation, such as that observed in intestinal mucositis, and for re-establishing homeostasis in patients undergoing chemo or radiotherapy.^11^ Probiotics are defined as “*live microorganisms that, when administered in adequate amounts, benefit the host*”,^12^ whereas postbiotics are inanimate microorganisms (*e.g.*, heat-inactivated bacterial cells) and/or their components (*e.g.*, cell-free bacterial culture supernatants) that confer health benefits.^13,14^ Among these, both probiotic and postbiotic *lactobacilli* have shown beneficial effects on intestinal homeostasis and are thus regarded as promising therapeutic agents for preventing disease development.^11,15,16^ Notably, current clinical practice guidelines from the Multinational Association of Supportive Care in Cancer and the International Society of Oral Oncology (MASCC/ISOO) support the use of probiotics containing *Lactobacillus* strains for the prevention of gastrointestinal mucositis induced by chemoradiotherapy, based on clinical evidence demonstrating efficacy in reducing severity and incidence.^17^ However, despite these recommendations, experimental studies exploring strain-specific effects and underlying mechanisms remain scarce, which are essential for optimizing probiotic interventions.

The species *Lacticaseibacillus rhamnosus* (formerly *Lactobacillus rhamnosus*) was recently reclassified following the taxonomic revision of the former broad *Lactobacillus* genus.^18^ Strains of *L. rhamnosus* have garnered attention for their potential to modulate intestinal inflammation and tissue damage.^19,20^ Among these strains, the *L. rhamnosus* GG strain (hereafter referred to as LGG), originally isolated from the healthy human intestine, is one of the most extensively studied probiotics and serves as a model for investigating the mechanisms underlying probiotic effects.^21‐23^ A study in mice revealed that high doses of LGG restored responsiveness to exogenous leptin, increased the villus-height-crypt depth ratio, and reduced the proportion of *Proteobacteria* in the gut microbiota.^24^ Additionally, LGG improved survival rates in septic C57BL/6 mice by promoting cell proliferation and reducing apoptosis.^25^ Recent studies have further emphasized the therapeutic potential of LGG both *in vitro* and *in vivo*. *In vitro* experiments have demonstrated the strain's efficacy against pathogen infections and intestinal inflammation.^15^ Additionally, LGG modulated the inflammatory response in 5-FU-treated Caco-2 cells by up-regulating increasing TNF-*α*, MCP-1, and IL-12 expression, highlighting its potential impact on chemotherapy-induced mucositis.^26^ Furthermore, treatment with LGG-fermented milk reduces inflammation and colonic lesions induced by DSS in mice.^27^ Neonatal colonization of mice with LGG promoted increased IgA production during development and adulthood, benefiting intestinal microbiota maturation and preventing DSS-induced intestinal lesions and inflammation.^28^

Other strains may possess probiotic properties that are both relevant and complementary to those of this reference strain.^29,30^ Among them, *L. rhamnosus* L156.4, a strain isolated from the feces of NIH mice, has demonstrated promising potential for animal and human health, notably through its ability to inhibit the growth of pathogenic bacteria.^31,32^ Given recognizing that the effects of probiotics are strain-specific, this work investigated the *in vivo* therapeutic effects of *L. rhamnosus* L156.4 administered as live cells, heat-inactivated cells and cell-free supernatant, in a murine model of intestinal mucositis induced by 5-fluorouracil (5-FU). Furthermore, we compared these effects with those of the well-known probiotic LGG.

## Materials and methods

### Microorganisms and growth conditions

*L. rhamnosus* L156.4 (hereafter named L156.4) was obtained from Taconic (Germantown, USA). LGG (ATCC 53103) was isolated from the commercial Culturelle Digestive Daily Probiotic Capsules© supplement, recovered as single colonies on MRS agar, and confirmed as *Lacticaseibacillus rhamnosus* by MALDI-TOF mass spectrometry. The bacterial stock cultures were stored at −80 °C in de Man, Rogosa, and Sharpe (MRS) broth (Sigma, United States of America) with 15% glycerol. The bacterial strains were cultured in MRS supplemented with Tween 80 (0.1% v/v) (Synth, Brazil) in a bacteriological incubator at 37 °C for 16 h with reduced headspace (low-oxygen environment). After growth, bacterial concentrations were standardized to 1 × 10⁹ colony-forming units (CFU)/mL, corresponding to an optical density (OD) of 1.2 at 600 nm for all preparations. To prepare the live bacterial suspensions, 1 mL of bacterial culture adjusted to an OD_600_ of approximately 1.2 (~1 × 10⁹ CFU/mL) was centrifuged at 13,000 rpm for 10 min. The pellet was washed twice with sterile 0.1 M phosphate-buffered saline (PBS; pH 7.0) and resuspended in 200 μL of sterile PBS. Thus, each daily 200 µL gavage delivered approximately 1 × 10⁹ CFU. These counts were validated by plating serial dilutions to confirm strain viability and the OD–CFU correlation. Live bacterial suspensions were inactivated by heating in a water bath at 75 °C for 15 min to prepare the heat-inactivated bacterial suspensions. The same bacterial suspensions used for the live preparations (OD_600_ ≈ 1.2, corresponding to ~1 × 10⁹ CFU/mL) were subjected to heat treatment to ensure equivalent bacterial concentration across treatments. Complete inactivation was confirmed by the absence of growth on MRS agar plates after 48 h of incubation at 37 °C. To prepare the bacteria-free supernatant suspensions, bacterial cultures were centrifuged at 13,000 rpm for 10 min and filtered through a 0.22 μm membrane filter, and the resulting supernatant was collected and stored at −80 °C until use. A MRS broth control was prepared under the same conditions (centrifuged and filtered). The pH values measured before storage were 5.90 for the MRS control, 4.10 for L156.4, and 3.89 for LGG.

### Animals

Male BALB/c mice, 7–8 weeks old, were obtained from the Federal University of Minas Gerais animal facility. Animals were maintained in ventilated mini-isolators under controlled temperature (25 ± 2 °C), and a 12 h light/dark cycle, with free access to water and food. Each cage housed seven animals from the same experimental group, with no co-housing or mixing across groups. Animal manipulation was carried out in compliance with the Brazilian National Council for the Control of Animal Experimentation—CONCEA (available at http://www.mctic.gov.br/concea) and was approved by the Ethics Committee in Animal Experimentation (CEUA/UFMG, Protocol 135/2024). Animals were randomly allocated to the experimental groups by simple lottery 7 d before the beginning of the treatments. Sample size was determined a priori based on a 25% variability for ileal gene expression,^33^ a 95% confidence level, and 95% power, resulting in an estimated eight animals per group. For feasibility, seven animals per group were used, maintaining adequate statistical power and complying with CEUA ethical guidelines.

### Experimental design

**Figure 1.**
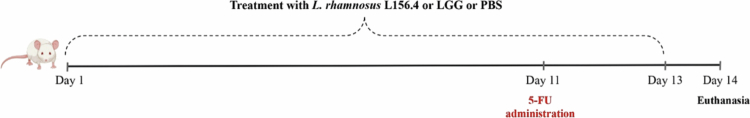
Experimental design to evaluate the effects of live, culture supernatant, and heat-inactivated *L. rhamnosus* strains L156.4 and GG in a murine model of intestinal mucositis induced by 5-FU. Mice (*n* = 7 per group for each experiment) were subjected to daily administration of bacterial preparations for 13 d, followed by mucositis induction on 11th day with a single intraperitoneal injection of 5-FU (300 mg/kg). On 14^th^ day, mice were euthanized for tissue collection and further analysis. The negative control group received an injection of PBS.

A series of three experiments were conducted to evaluate the effects of (i) live L156.4 and LGG strains, (ii) heat-inactivated L156.4 and LGG strains, and (iii) L156.4 and LGG culture supernatants on intestinal mucositis in mice. For each experiment, mice were divided into four groups (*n* = 7 animals per group) each housed in a single ventilated cage, with no co-housing or mixing of animals from different groups throughout the pretreatment and treatment periods. as described below: negative control group (NC), 5-FU mucositis group (MUC), 5-FU mucositis group treated with L156.4-derived preparations (*i.e.*, live L156.4 [L156.4 MUC], heat-inactivated L156.4 [iL156.4 MUC], or L156.4 culture supernatant [sL156.4 MUC]), and 5-FU mucositis group treated with LGG-derived preparations (*i.e.*, live LGG [LGG MUC], heat-inactivated LGG [iLGG MUC], or LGG culture supernatant [sLGG MUC]). The bacterial preparations (200 µL) were administered once daily by oral gavage for 13 consecutive days (Figure 1). Treatment with the bacterial preparations (*i.e.*, live, heat-inactivated, or supernatant forms of strains L156.4 and LGG) began 10 d prior to 5-FU injection (Day 11) and continued for 48 h following the induction of mucositis, with the final administration occurring on Day 13. On Day 14, no treatment was given, as animals underwent DTPA intestinal permeability assays and were subsequently euthanized for tissue and sample collection. The negative control group (NC) and the positive mucositis group (MUC) received 200 μL of PBS over the same period. Mucositis was induced in the treatment and positive control groups *via* a single intraperitoneal injection of 5-fluorouracil (5-FU) at a dose of 300 mg/kg (Flusan®, Eurofarma, São Paulo, SP, Brazil), whereas the negative control group received 100 µL of PBS intraperitoneally, as represented in Figure 1. At 72 h post-mucositis induction, all the animals were anesthetized with a ketamine (80 mg/kg) and xylazine (16 mg/kg) mixture solution and euthanized by cervical dislocation. The ileum section was subsequently collected for further analysis.

### Body weight loss and intestinal length

The mice were weighed daily simultaneously on a 0.25–2.000 g precision balance. Weight loss (%) was calculated after the induction of mucositis. The entire small intestine was measured to assess intestinal length, and the results are expressed in centimeters.

### Intestinal permeability

Intestinal permeability (IP) was measured according to the methodology previously described by^34^. Briefly, all animals received by gavage 0.1 mL of diethylenetriaminepentaacetic acid radiolabeled with technetium-99m solution (99mTc-DTPA) containing 18.5 MBq of radioactivity. After 4 h, mice were anesthetized, and 300 µL of blood were collected and placed in appropriate tubes for the determination of radioactivity using a gamma counter (Wizard 1470, PerkinElmer, Waltham, MA, USA). A dose standard containing the same radioactivity level was administered for all animals and employed for decay correction of technetium-99m. The IP was expressed as the percentage of the radiation dose in the blood, calculated as % dose administered/g = (cpm per gram of blood/cpm of standard) × 100. The radiation activity administered was 3.7 MBq (MegaBequerel) per animal.

### Histomorphological analysis and goblet cell count

Ileum samples were collected after euthanasia, rinsed in 0.1 M phosphate-buffered saline (PBS), rolled, and transferred to histological cassettes. Samples were fixed using 10% neutral buffered formalin, a solution containing 40% formaldehyde, 0.4% sodium dihydrogen phosphate, and 0.65% disodium hydrogen phosphate. The tissues were subsequently embedded in paraffin, sectioned into 4 μm slices, and mounted onto glass slides for staining with hematoxylin and eosin (H&E) or periodic acid–Schiff (PAS), following the procedure described by^33^. To assess mucosal injury, a modified version of the scoring system proposed by^35^ was used, evaluating five histopathological criteria: villus atrophy, brush border disruption, crypt architectural damage, infiltration of inflammatory cells, and edema of the submucosal and muscular layers. A grading scale from 0 (absence of damage) to 3 (intense damage) was applied to each criterion, and individual scores were totaled per animal. The specific criteria and scoring rubric are summarized in Table 1. All slides were coded, and histopathological scoring was performed by a blinded evaluator who was unaware of group assignments. Morphological measurements were obtained from randomly selected sections using an Olympus BX41 microscope equipped with a 20× objective, assessing 20 villi and 20 crypts per animal. Goblet cell quantification was carried out on PAS-stained slides by counting 10 random fields per slide under 40× magnification. All image-based measurements were processed using ImageJ (NIH, Bethesda, MD, USA), and data were expressed as mean values for each experimental group.

**Table 1.** Histopathological scoring criteria for intestinal injury according to the modified^35^ scale.

Criterion	Description	Score range
Villus atrophy	Reduction in villus height and surface area	0–3
Brush border disruption	Loss or fragmentation of enterocyte microvilli	0–3
Crypt architectural damage	Shortening, disorganization, or epithelial loss	0–3
Inflammatory cell infiltration	Presence and extent of mucosal or submucosal infiltrate	0–3
Submucosal/muscular edema	Interstitial swelling in the submucosa or muscular layers	0–3

### Myeloperoxidase activity assay

Myeloperoxidase (MPO) activity, an indirect marker of neutrophil infiltration in intestinal tissue, was measured, with modifications.^36,37^ Briefly, 100 mg of ileum were homogenized in a buffer containing 0.1 M NaCl and 0.015 M Na₂EDTA (pH 4.7), followed by sequential centrifugation steps, lysis with 0.5% hexadecyltrimethylammonium bromide (HTAB), and three freeze‒thaw cycles in liquid nitrogen. Enzyme activity was quantified through a colorimetric reaction using TMB as substrate, and absorbance was read at 450 nm. Results are expressed as absorbance-based arbitrary units (A_450_ per mg of tissue), as no calibration curve was generated. This approach, widely applied in experimental models of intestinal inflammation, allows reliable comparison of relative MPO activity between groups.

### RNA extraction and real-time qPCR analyzes

RNA was isolated from the distal ileum section using the Pure Link™ RNA Mini Kit (Invitrogen, Carlsbad, USA) by the manufacturer's protocol. The integrity of the RNA was evaluated using a 1.5% agarose gel. A total of 2 μg of RNA, quantified with a NanoDrop 2000 spectrophotometer (Thermo Scientific, Waltham, USA), was reverse transcribed into complementary DNA (cDNA) using the High-Capacity cDNA Reverse Transcription Kit (ThermoFisher, Waltham, USA) following the manufacturer's instructions. Quantitative PCR (qPCR) was performed using the PowerUp™ SYBR® Green Master Mix (ThermoFisher) on the ABI PRISM 7900HT Sequence Detection System (Applied Biosystems™). The thermal cycling conditions were as follows: 95 °C for 10 min, followed by 40 cycles of 95 °C for 15 s and 60 °C for 1 min. All primers used were described in Table 2. Primer pair efficiency and specificity were assessed, with melting curve analysis confirming the amplification of a single PCR product and efficiencies ranging from 90% to 110%. Relative gene expression levels were normalized to the geometric mean of three reference genes: *Actb* (actin beta), *Gapdh* (glyceraldehyde-3-phosphate dehydrogenase), and *Ppia* (peptidyl-prolyl isomerase A). The online tool RefFINDER (https://www.ciidirsinaloa.com.mx/RefFinder-master/)^38^ was used to identify these genes as the most stably expressed genes in the experimental conditions tested from a panel of five candidate genes. Gene expression values were calculated using the 2^−ΔΔCT^ method relative to the control condition (*i.e*., negative control group) and expressed as mean ± SD.^39^

**Table 2.** Primers used in the RT-qPCR assays.

Gene name	Product	Primer pair sequence	Reference
Actb*	Actin beta	5′-GCTGAGAGGGAAATCGTGCGTG -3′/ 5′-CCAGGGAGGAAGAGGATGCGG-3′	[40]
Gapdh*	Glyceraldehyde-3-phosphate dehydrogenase	5′-TCACCACCATGGAGAAGGC -3′/ 5′-GCTAAGCAGTTGGTGGTGCA-3′	[41]
Ppia*	Peptidyl-prolyl isomerase A	5′-GGCAAATGCTGGACCAAAC-3′/ 5′-CATTCCTGGACCCAAAACG-3′	[42]
*Cldn1*	Claudin 1	5′-GTCATCGCCCATCAGAAGAT-3′/ 5′-ACTGTTGGACAGGGAACCAG-3′	[40]
*Il10*	Interleukin-10	5′-GGTTGCCAAGCCTTATCGGA-3′/ 5′-ACCTGCTCCACTGCCTTGCT-3′	[41]
*Il1b*	Interleukin-1 beta	5′-CTCCATGAGCTTTGTACAAGG-3′/ 5′-TGCTGATGTACCAGTTGGGG-3′	[43]
*Muc2*	Mucin 2	5′-GATGGCACCTACCTCGTTT-3′/ 5′-GTCCTGGCACTTGTTGGAAT-3′	[40]
*Nfkb1*	Nuclear factor kappa B subunit 1	5′- GTGGAGGCATGTTTCGGTAGTG-3′/ 5′- TCTTGGCACAATCTTTAGGGC-3′	[44]
*Ocln*	Occludin	5′-ACTCCTCCAATGGACAAGTG-3′/ 5′-CCCCACCTGTCGTGTAGTCT-3′	[40]
*Tgfb1*	Transforming growth factor beta 1	5′-TGACGTCACTGGAGTTGTACGG-3′/ 5′-GGTTCATGTCATGGATGGTGC-3′	[41]
*Tjp1*	Tight junction protein 1	5′-CCACCTCTGTCCAGCTCTTC-3′/ 5′-CACCGGAGTGATGGTTTTCT-3′	[40]
*Tnf*	Tumor necrosis factor	5′-ACGTGGAACTGGCAGAAGAG-3′/ 5′-CTCCTCCACTTGGTGGTTTG-3′	[43]

* Reference genes.

### Fecal DNA isolation and sequencing

Metagenomic DNA was extracted from 28 mouse fecal samples using the QIAamp DNA Stool Mini Kit (Qiagen), following the manufacturer's protocol, as described by^45^, with minor adjustments. Shotgun libraries were prepared using the TruSeq DNA Sample Preparation Kit (Illumina), and sequencing was conducted on the Illumina HiSeq 2500 platform, generating paired-end reads (2 × 150 bp) with an average insert size of 450 bp.

### Microbiome taxonomy and diversity analysis

The integrity and base quality of the raw sequencing reads were assessed using FastQC v0.11.9. Reads were quality-filtered using fastp v0.23.2, removing bases/reads with Phred scores below 30. Host-derived reads were removed by mapping the quality-filtered reads against the C57BL/6 mouse reference genome assembly GRCm39 using Bowtie2 v2.4.1, and only the non-host reads were retained for downstream analyzes. Taxonomic assignment of the remaining 150 bp paired-end reads was then performed with Kraken2 v2.1.3 using the built-in STANDARD database. To account for uneven sequencing depth across samples rarefaction curves were generated to assess sequencing depth sufficiency. Total Sum Scaling (TSS) normalization was applied to preserve all sequencing data while mitigating the influence of variable library sizes.^46^ All microbiome data were analyzed using RStudio integrated functions and specific packages. Differential abundance analysis was performed using the ALDEx2 package through its high-level aldex() function. Centered log-ratio (CLR) values were estimated from Monte Carlo Dirichlet instances (mc.samples = 128, denom = “all”), and statistical significance across experimental groups was assessed using the Kruskal–Wallis test implemented internally in ALDEx2 (test = “kw”), which provides an ANOVA-like comparison suitable for compositional microbiome data. False discovery rate (FDR) correction was applied using the Benjamini–Hochberg method on within-effect *p*-values. Microbial community structure was assessed using Principal Coordinates Analysis (PCoA) based on Bray–Curtis dissimilarity matrices calculated from normalized abundance data. Microbial community metrics were then estimated from the TSS-normalized data, including diversity (Shannon), richness (Chao1), and evenness (Pielou), using thevegan package in R.^47‐49^

### Statistical analysis

For clinical parameters and host response markers, statistical analyzes were performed using GraphPad Prism 10.2.0 (San Diego, CA, USA). Data normality was assessed using the Shapiro–Wilk test. Non-parametric datasets were analyzed using Kruskal–Wallis followed by Dunn's post hoc test, whereas normally distributed datasets were evaluated using one-way ANOVA followed by Tukey's post hoc test. In cases where there was unequal variance (heteroscedasticity) between groups, the Brown–Forsythe or Welch ANOVA were used, depending on the data characteristics (test results are summarized in Table S1). For microbiome data, all analyzes were conducted in RStudio v.4.5.0. Relative abundances at the phylum and genus levels were CLR-transformed and compared among groups using Welch's t-tests, with *p*-values adjusted using the Benjamini–Hochberg false discovery rate (FDR) correction. For beta diversity (PCoA), group separation was tested by PERMANOVA (adonis2, 999 permutations), and homogeneity of multivariate dispersion was evaluated using the betadisper function from the *vegan* package. Alpha-diversity metrics were calculated using TSS-normalized data and compared using the same statistical approaches applied to clinical parameters. All statistical results for each comparison, including *p*-values and effect sizes, are provided in Supplementary Material 2. Statistical significance was defined as *p* < 0.05.

## Results

### Preventive treatment with live L156.4 and LGG reduces neutrophilic infiltration and attenuates 5-FU–induced intestinal injury

**Figure 2.**
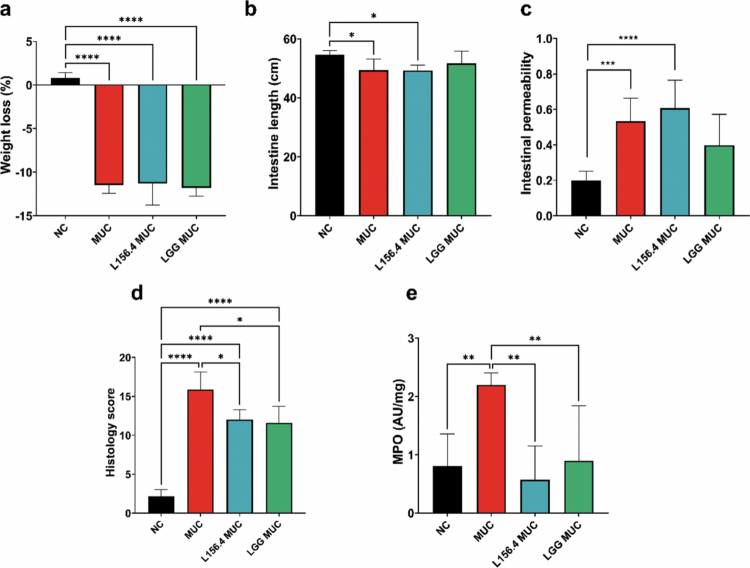
Effects of daily administration of live *L. rhamnosus* strains L156.4 and GG on chemotherapy-induced mucositis in mice. Weight loss (a), small intestine length (b), intestinal permeability (c), histopathology score (d), and MPO activity in ileum tissue (e) were evaluated. NC, negative control group; MUC, mucositis control group; L156.4 MUC, mucositis group treated with L156.4; LGG MUC, mucositis group treated with LGG. Statistical analysis consisted of ordinary one-way ANOVA followed by Tukey's multiple comparisons test for weight loss (a), intestine length (b), and histopathology score (d); Kruskal–Wallis test followed by Dunn's multiple comparisons test for intestinal permeability (c); and Brown–Forsythe and Welch ANOVA tests followed by Dunnett's T3 multiple comparisons test for MPO activity (e). Statistical differences: *p* < 0.05 (*), p < 0.01 (**), p < 0.001 (***), *p* < 0.0001 (****).

The first experiment aimed to evaluate the protective effects of daily administration of live L156.4 and LGG in the context of 5-FU-induced intestinal mucositis in a mouse model. Following 70 h of 5-FU administration, mice in the mucositis group (MUC) exhibited pronounced alterations compared with the control group (NC), characterized by significant body weight loss (*p = 4.5 × 10* ^*−13*^) and marked shortening of the small intestine (*p = 2.53 × 10^−^* ^*2*^), indicative of 5-FU–induced structural damage (Figure 2a and b). Intestinal barrier integrity was further impaired, as shown by a significant increase in intestinal permeability (*p = 5.5 × 10^−^* ^*3*^) and histological damage scores (*p = 1.0 × 10^−^* ^*12*^) compared with NC (Figure 2c and d). Finally, neutrophil infiltration, assessed by myeloperoxidase (MPO) activity, was significantly higher, indicating a pronounced inflammatory response induced by 5-FU (*p = 1.5 × 10^−^* ^*3*^) (Figure 2e). Treatment with either L156.4 or LGG did not significantly improve body weight loss or intestinal length, nor did it restore intestinal permeability, which remained comparable to that observed in the MUC group (Figure 2a–c). In contrast, both probiotic strains significantly reduced histological damage scores compared with MUC mice (*p = 2.0 × 10^−^* ^*3*^ for L156.4 and *p = 6.5 × 10^−^* ^*4*^ for LGG) (Figure 2d). Moreover, L156.4 and LGG markedly decreased MPO activity to levels comparable to those of the NC group (*p = 8.7 × 10^−^* ^*3*^ and *p = 4.58 × 10^−^* ^*2*^, respectively), indicating attenuation of neutrophil-driven inflammation (Figure 2e).

**Figure 3.**
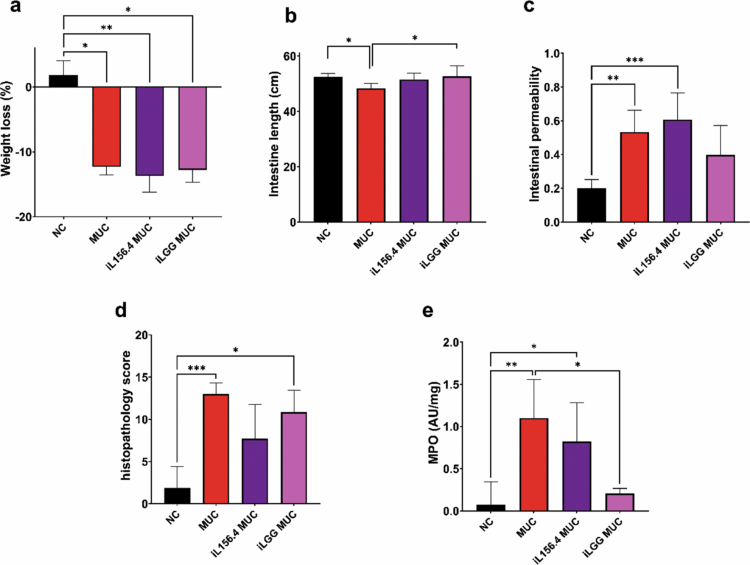
Effects of daily administration of heat-inactivated *L. rhamnosus* strains L156.4 and GG on chemotherapy-induced mucositis in mice. Weight loss (a), small intestine length (b), intestinal permeability (c), histopathology score (d), and MPO activity in ileum tissue (e) were evaluated. NC, negative control group; MUC, mucositis control group; iL156.4 MUC, mucositis group treated with heat-inactivated L156.4; iLGG MUC, mucositis group treated with heat-inactivated LGG. Statistical analysis consisted of Kruskal–Wallis test followed by Dunn's multiple comparisons test for weight loss (a), intestinal permeability (c), and histopathology score (d); ordinary one-way ANOVA followed by Tukey's multiple comparisons test for intestine length (b); and Brown–Forsythe and Welch ANOVA tests followed by Dunnett's T3 multiple comparisons test for MPO activity (e). Statistical differences: *p* < 0.05 (*), p < 0.01 (**), p < 0.001 (***), *p* < 0.0001 (****).

The second experiment aimed to evaluate the effects of heat-inactivated L156.4 and LGG in preventing 5-FU-induced damage. The MUC group displayed typical mucositis damage (Figure 3). While both treatments failed to prevent weight loss (Figure 3a), only heat-inactivated LGG partially preserved small intestinal length, with a significant increase compared with MUC *(p = 1.92 × 10^−^* ^*2*^ *)*, reaching values close to those of NC (Figure 3b). Neither L156.4 nor LGG treatments significantly reduced intestinal permeability or histological scores (Figure 3c and d). Finally, treatment with heat-inactivated LGG significantly reduced MPO activity, suggesting a role in modulating the inflammatory response (*p = 1.09 × 10^−^* ^*2*^) (Figure 3e).

**Figure 4.**
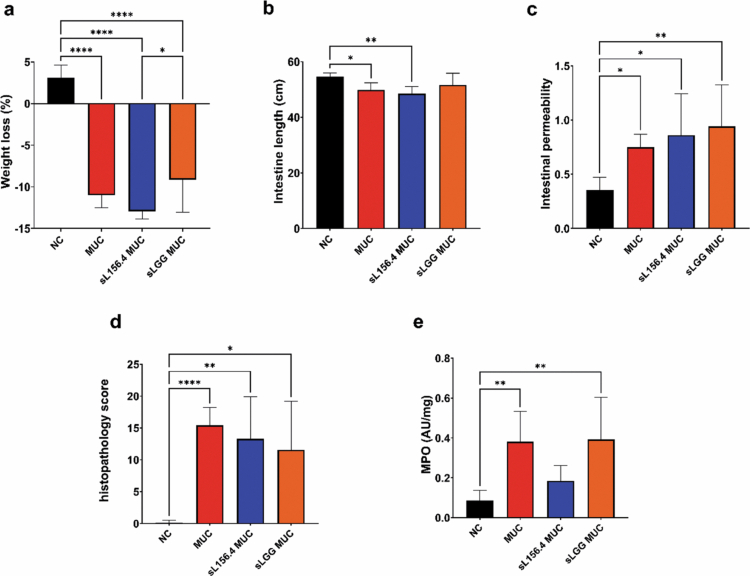
Effects of daily administration of *L. rhamnosus* strains L156.4 and GG culture supernatant on chemotherapy-induced mucositis in mice. Weight loss (a), small intestine length (b), intestinal permeability (c), histopathology score (d), and MPO activity in ileum tissue (e) were evaluated. NC, negative control group; MUC, mucositis control group; sL156.4 MUC, mucositis group treated with L156.4 culture supernatant; sLGG MUC, mucositis group treated with LGG culture supernatant. Statistical analysis consisted of ordinary one-way ANOVA followed by Tukey's multiple comparisons test for weight loss (a), intestine length (b), and MPO activity (e); Kruskal–Wallis test followed by Dunn's multiple comparisons test for intestinal permeability (c); and Brown–Forsythe and Welch ANOVA tests followed by Dunnett's T3 multiple comparisons test for histopathology score (d). Statistical differences: *p* < 0.05 (*), p < 0.01 (**), p < 0.001 (***), *p* < 0.0001 (****).

The third experiment demonstrated that daily administration of L156.4 and LGG culture supernatants was ineffective in preventing the adverse effects induced by 5-FU. As previously observed, the MUC group showed significant reductions in body weight and small intestine length (*p = 1.62 × 10* ^*−10*^ and *p = 3.22 × 10^−^* ^*2*^, respectively) (Figure 4a and b), along with increased intestinal permeability, histological scores, and MPO activity (*p = 1.87 × 10^−^* ^*2*^, *p = 3.47 × 10^−5^*, and *p = 3.7 × 10^−^* ^*3*^, respectively) (Figure 4c, d, and e) compared to the NC group. All parameters in the groups treated with bacterial culture supernatants were similar to those in the MUC group, with no statistically significant differences (Figure 4).

Based on the results obtained, the administration of live L156.4 and LGG represents a promising strategy to prevent 5-FU-induced intestinal mucositis in an experimental mouse model. Thus, further analyzes using live bacterial preparations were conducted to elucidate the mechanisms of action involved.

### Treatment with live L156.4 and LGG partially preserves intestinal architecture and the goblet cell population

**Figure 5.**
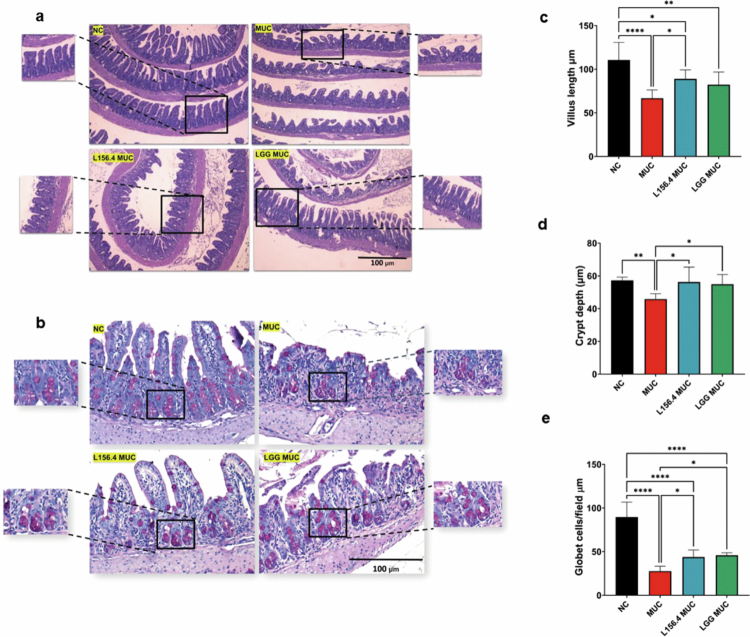
Effects of daily administration of live *L. rhamnosus* strains L156.4 and GG on intestinal tissue architecture in mice with chemotherapy-induced mucositis. Representative photomicrographs of intestinal tissue stained with H&E (a) and PAS (b) are shown (scale bar = 100 µm). Morphometric analyzes of villus length (c), crypt depth (d), and goblet cells per field (e) were performed. NC, negative control group; MUC, mucositis control group; L156.4 MUC, mucositis group treated with L156.4; LGG MUC, mucositis group treated with LGG. Statistical analysis consisted of ordinary one-way ANOVA followed by Tukey's multiple comparisons test for all variables (*villus length, crypt depth,* and *goblet cells/field*). Statistical differences: *p* < 0.05 (*), p < 0.01 (**), p < 0.001 (***), *p* < 0.0001 (****).

Figure 5a and b illustrates the structural alterations in the intestinal mucosa and the goblet cell population induced by administering 5-FU in mice. Compared with the NC group, the MUC group presented a significant reduction in villus length (*p = 3.21 × 10^−5^*) (Figure 5c), crypt depth *(p = 9.50 × 10^−3^*) (Figure 5d), and goblet cell count (*p = 1.53 × 10^−10^*) (Figure 5e), indicating pronounced deterioration of intestinal architecture. Preventive treatment with live L156.4 significantly counteracted the negative impact of 5-FU administration on villus length (*p = 3.38 × 10^−2^*), crypt depth (*p = 1.39 × 10^−2^*), and goblet cell count (*p = 2.88 × 10^−2^*) compared with the MUC group. In contrast, treatment with LGG had a limited effect, with improvements restricted to crypt depth and goblet cell count (*p = 3.78 × 10^−^* ^*2*^ and *p = 1.2 × 10^−^* ^*2*^, respectively) (Figure 5d and e).

### Treatment with live L156.4 and LGG modulates cytokine and barrier-related gene expression in a mucositis model

**Figure 6.**
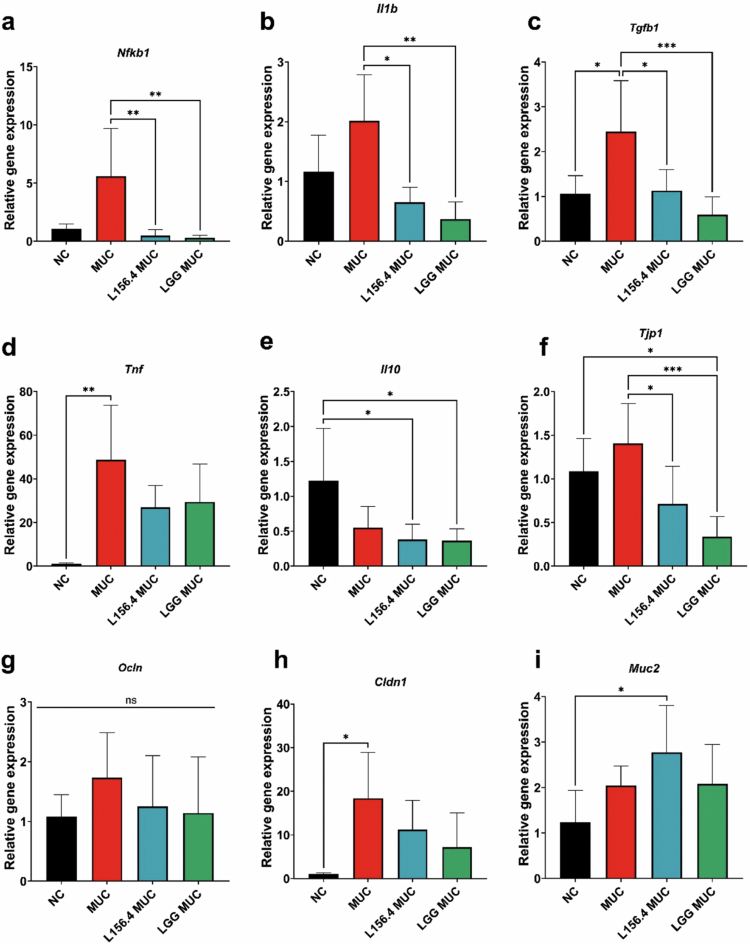
Effects of daily administration of live *L. rhamnosus* strains L156.4 and GG on inflammation- and epithelial barrier-related gene expression in mice with chemotherapy-induced mucositis. Relative expression levels of inflammation-related genes: (a) *Nfkb1* (nuclear factor kappa B subunit 1), (b) *Il1b* (interleukin 1 beta), (c) *Tgfb1* (transforming growth factor beta 1), (d) *Tnf* (tumor necrosis factor), and (e) *Il10* (interleukin 10). Relative expression levels of epithelial barrier-related genes: (f) *Tjp1* (tight junction protein 1), (g) *Ocln* (occludin), (h) *Cldn1* (claudin 1), and (i) *Muc2* (mucin 2). NC, negative control group; MUC, mucositis control group; L156.4 MUC, mucositis group treated with L156.4; LGG MUC, mucositis group treated with LGG. Statistical analysis consisted of ordinary one-way ANOVA followed by Tukey's multiple comparisons test for parametric data (*Il10, Tjp1, Ocln, Cldn1, Muc2, Tgfb1*), and the Kruskal–Wallis test followed by Dunn's multiple comparisons test for nonparametric data (*Nfkb1, Il1b, Tnf*). *p*-values were adjusted using the Bonferroni correction where applicable. Statistical differences: *p* < 0.05 (*), p < 0.01 (**), p < 0.001 (***), *p* < 0.0001 (****).

The expression of key inflammatory and barrier-related genes was analyzed to assess the cellular response and the impact of probiotic treatments on 5-FU-induced mucositis. Among the inflammatory marker genes (*i.e.*, *Nfkb1*, *Il1b*, *Tgfb1*, *Tnf*), MUC animals tended to show higher transcript levels than NC, and this increase reached statistical significance for *Tgfb1* and *Tnf* (*p = 2.4 × 10^−^* ^*2*^ and *p = 4.0 × 10^−^* ^*3*^ for NC *vs* MUC, respectively) (Figure 6a–d). Compared with the MUC group, *Nfkb1* (*p = 8.9 × 10^−^* ^*3*^ for L156.4 MUC, and *p = 2.6 × 10^−^* ^*3*^ for LGG MUC), *Il1b* (*p = 4.99 × 10^−^* ^*2*^ for L156.4 MUC, and *p = 1.8 × 10^−^* ^*3*^ for LGG MUC), and *Tgfb1* (*p = 1.07 × 10^−^* ^*2*^ for L156.4 MUC, and *p = 4.0 × 10^−^* ^*4*^ for LGG MUC) expression levels were significantly reduced in both probiotic-treated groups, returning to values comparable to those observed in NC mice (Figure 6a, b, and c). Although *Tnf* expression tended to be lower in the L156.4 and LGG groups than in MUC these differences did not reach statistical significance (Figure 6d). *Il10* expression did not differ significantly between NC and MUC animals; however, it was significantly decreased in the L156.4 MUC *(p = 1.33 × 10^−^* ^*2*^ *)* and LGG MUC *(p = 1.96 × 10^−^* ^*2*^ *)* groups compared with NC (Figure 6e). Among the barrier-related genes assessed (*Clnd1*, *Tjp1*, *Ocln*, *Muc2*), only *Clnd1* expression was significantly modulated in the MUC group compared with NC (*p = 1.01 × 10^−^* ^*2*^) (Figure 6h). In L156.4- and LGG-treated mice, *Cldn1* expression showed intermediate values that were lower than in MUC but did not differ significantly from either NC or MUC. *Ocln* expression remained unchanged across all groups (Figure 6g). In contrast, *Tjp1* expression was significantly affected by both 5-FU and probiotic treatment: it was lower in L156.4 MUC and LGG MUC than in MUC (*p = 2.3 × 10^−^* ^*2*^ and *p = 3.0 × 10^−^* ^*4*^, respectively), and LGG MUC also differed from NC (*p = 1.5 × 10^−^* ^*2*^), indicating a complex pattern of modulation rather than a simple restoration to baseline (Figure 6f). Finally, *Muc2* expression was significantly increased in the L156.4 MUC group compared with NC (*p = 1.91 × 10^−^* ^*2*^) (Figure 6i). Taken together, these results indicate that both L156.4 and LGG attenuate 5-FU-induced activation of NF-κB-related inflammatory genes and modulate barrier-associated transcripts, with L156.4 specifically increasing *Muc2* expression.

### Treatment with live LGG partially restores fecal microbiota diversity

**Figure 7.**
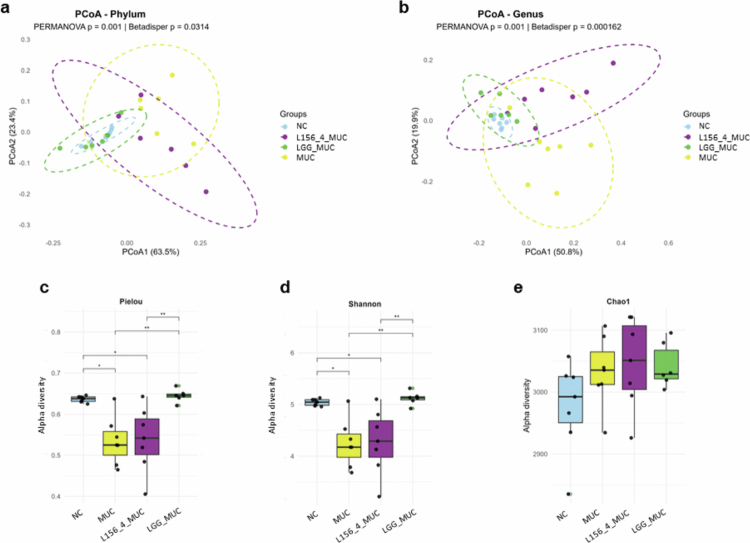
Effects of daily administration of live *L. rhamnosus* strains L156.4 and GG on fecal microbiota structure in mice with chemotherapy-induced mucositis. Principal coordinate analysis (PCoA) and alpha-diversity metrics of the fecal microbiota. PCoA plots of bacterial communities at the phylum (a) and genus (b) levels were generated using Bray–Curtis dissimilarity. Ellipses represent 95% confidence intervals for each group. Statistical significance of community structure was evaluated by PERMANOVA, and homogeneity of dispersion was assessed by betadisper. Evenness estimated by the Pielou index (c), microbial diversity by the Shannon index (d), and richness by the Chao1 index (c) are shown. NC, negative control group; MUC, mucositis control group; L156.4 MUC, mucositis group treated with L156.4; LGG MUC, mucositis group treated with GG. Statistical analysis consisted of ordinary one-way ANOVA followed by Tukey's multiple comparisons test for alpha-diversity metrics (*Chao1, Pielou,* and *Shannon*). Statistical differences: *p* < 0.05 (*), p < 0.01 (**), p < 0.001 (***), *p* < 0.0001 (****).

The impact of the treatments on the fecal microbiota of mice was assessed using beta-diversity, alpha-diversity, evenness, and richness analyzes. Beta diversity was assessed using principal coordinate analysis (PCoA) based on Bray–Curtis dissimilarity (Figure 7a and b). At the phylum level (Figure 7a), PCoA1 (63.5%) and PCoA2 (23.4%) revealed clear differences in community structure among groups. Samples from the MUC group clustered apart from the others, while LGG-treated mice grouped closely with the NC samples. The L156.4-treated group showed an intermediate position between NC and MUC, indicating partial modulation of the microbial profile. These differences were confirmed by PERMANOVA (*p = 1.0 × 10^−^* ^*3*^ *, R² = 0.23*), although the betadisper test indicated heterogeneous dispersion among groups (*p = 3.14 × 10^−^* ^*2*^), which should be considered when interpreting group separation. At the genus level (Figure 7b), PCoA1 (50.8%) and PCoA2 (19.9%) similarly separated the groups, with MUC samples remaining distinct and more dispersed, whereas LGG MUC samples closely overlapped with NC, reinforcing that LGG exerted a stronger restorative effect than L156.4 on the overall microbial composition. Significant group-level differences were also detected by PERMANOVA (*p = 1.0 × 10^−^* ^*3*^) and betadisper (*p = 1.62 × 10^−^* ^*4*^) analyzes.

Alpha diversity and evenness analyzes supported these findings (Figure 7c–e). The MUC group showed significantly reduced Pielou evenness (*p = 5.5 × 10^−^* ^*3*^) and Shannon diversity (*p = 6.7 × 10^−^* ^*3*^) compared with NC, whereas LGG-treated mice restored both indices to NC levels (*p = 0.99* for Pielou; *p = 0.98* for Shannon). L156.4-treated animals showed only a partial recovery, without statistical significance (*p = 0.999 vs* MUC for both indices). Richness (Chao1) did not differ among groups, indicating that the main impact of mucositis and probiotic treatment was on community balance and diversity, rather than species number.

### Treatment with live L156.4 and LGG modulate key gut bacterial phylum and genera composition

**Figure 8.**
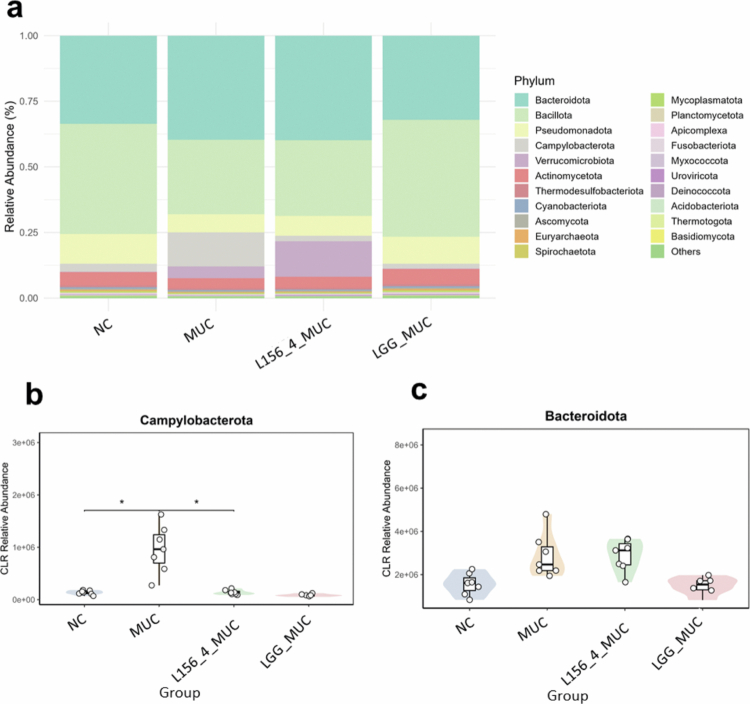
Effects of daily administration of live *L. rhamnosus* strains L156.4 and GG on fecal microbiota composition at the phylum level in mice with chemotherapy-induced mucositis. Relative abundance of bacterial phyla (a), *Campylobacterota* (b), and *Bacteroidota* (c) are shown. NC, negative control group; MUC, mucositis control group; L156.4 MUC, mucositis group treated with L156.4; LGG MUC, mucositis group treated with GG. The results indicate significant modulation of the intestinal microbiota following therapeutic interventions. Statistical analysis consisted of the Kruskal–Wallis test followed by Welch's *t*-test with Benjamini–Hochberg false discovery rate (FDR) correction for all comparisons. Statistical differences: *p* < 0.05 (*), p < 0.01 (**), p < 0.001 (***), *p* < 0.0001 (****).

**Figure 9.**
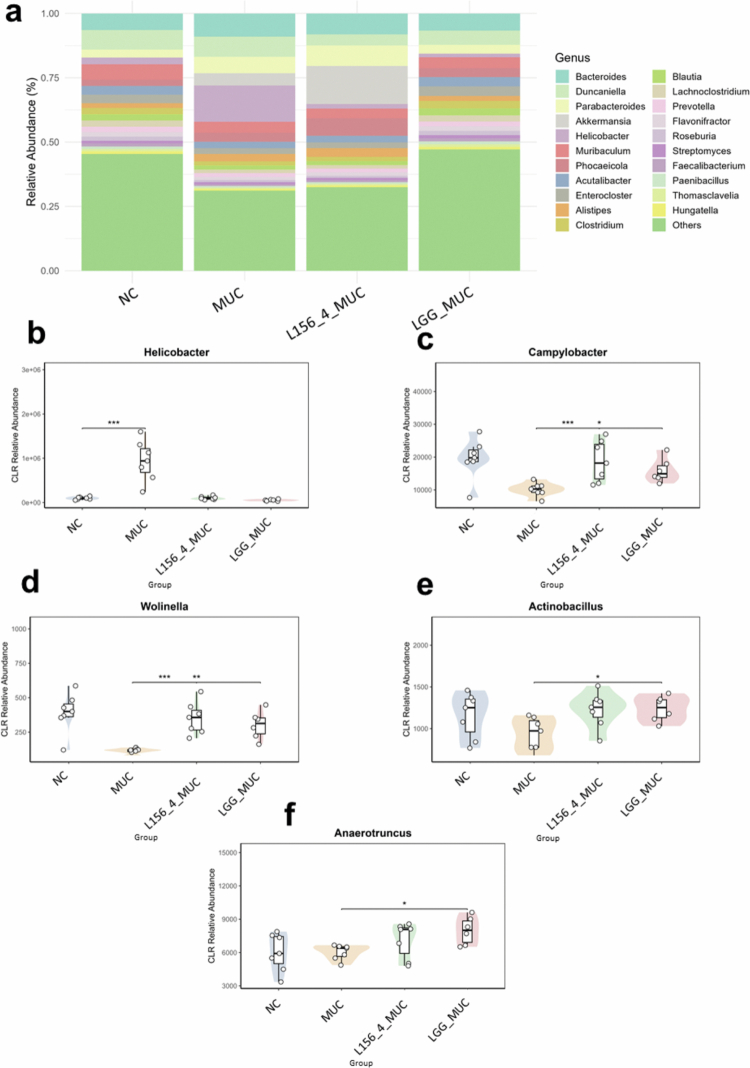
Effects of daily administration of live *L. rhamnosus* strains L156.4 and GG on fecal microbiota composition at the genus level in mice with chemotherapy-induced mucositis. Relative abundance of bacterial genera (a), *Helicobacter* (b), *Campylobacter* (c), *Wolinella* (d), *Actinobacillus* (e), and *Anaerotruncus* (f) are shown. NC, negative control group; MUC, mucositis control group; L156.4 MUC, mucositis group treated with L156.4; LGG MUC, mucositis group treated with GG. The results indicate significant modulation of the intestinal microbiota following therapeutic interventions. Statistical analysis consisted of the Kruskal–Wallis test followed by Welch's *t*-test with Benjamini–Hochberg false discovery rate (FDR) correction for all comparisons. Statistical differences: *p* < 0.05 (*), p < 0.01 (**), p < 0.001 (***), *p* < 0.0001 (****).

The impact of the treatments on the fecal microbiota composition was also assessed at the phylum and genus levels (Figures 8 and 9). The stacked bar plots illustrate the relative abundance of dominant bacterial phyla (Figure 8a) and genera (Figure 9a) across the experimental groups. As expected, MUC mice showed marked changes in community structure relative to NC animals. At the phylum level, microbiota was characterized by a significant increase in *Campylobacterota (p = 2.34 × 10^−^* ^*2*^ *)* (Figure 8b), whereas changes in *Bacteroidota*, *Verrucomicrobiota*, *Bacillota* and *Pseudomonadota* did not reach statistical significance (Figure 8c, and +e S2). At the genus level, MUC mice exhibited a significant increase in inflammation-associated taxa such as *Helicobacter*, *Desulfobacter* and *Pulveribacter*, while non-significant differences were observed for several other genera, including *Bacteroides*, *Parabacteroides*, *Akkermansia*, *Campylobacter*, *Wolinella* and *Actinobacillus* (Figure 9b–f, and Table S3). LGG supplementation appeared to partially mitigate 5-FU–induced alterations, shifting the microbiota profile toward that of the NC group (Figures 8a and 9a). Significant changes were detected mainly in subdominant genera, including, among others, *Campylobacter (p = 3.64 × 10^−^* ^*2*^ *)*, *Wolinella (p = 1.9 × 10^−^* ^*3*^ *)*, *Actinobacillus (p = 1.8 × 10^−^* ^*2*^ *)*, and *Lacticaseibacillus (p = 1.0 × 10^−^* ^*3*^ *).* Although the overall microbiota profile of L156.4-treated mice remained more similar to that of the MUC group than to NC animals (Figures 8a and 9a), L156.4 nevertheless induced a significant reduction (*p = 4.65 × 10^−^* ^*2*^) in *Campylobacterota* abundance, a change not significant with LGG. In addition, L156.4 modulated several subdominant genera, similarly to LGG, shifting their relative abundances toward levels comparable to those of the NC group (Figure 9, and Table S3).

## Discussion

Probiotics have been widely studied for their potential to mitigate chemotherapy-induced mucositis by modulating inflammation and the gut microbiota.^50‐52^ Chemotherapy-induced mucositis is a multifactorial process involving direct epithelial and stem-cell injury, activation of stress and danger pathways, amplification of inflammatory cascades, ulceration, and subsequent healing.^53,54^ In this study, we evaluated the efficacy of a newly isolated murine strain, *L. rhamnosus* L156.4, in comparison with the well-established *L. rhamnosus* GG in a severe 5-FU induced mucositis model. In addition to live preparations, we also evaluated postbiotic fractions from these two strains, namely heat-inactivated bacteria and bacterial culture supernatants. This model reproduced key features of small-intestinal mucositis, including body weight loss, intestinal shortening, marked histological damage, increased permeability, and dysbiosis, capturing the peak inflammatory phase at 72 h, and provided a framework to compare the preventive effects of live and postbiotic preparations of L156.4 and LGG.

At the structural level, mucositis induced villus atrophy, decreased crypt depth, and a pronounced loss of goblet cells, consistent with disruption of both absorptive and secretory compartments. Live L156.4 and LGG partially preserved mucosal architecture, lowering histological scores and improving crypt depth and goblet cell counts, with L156.4 also exerting a clearer effect on villus length. These changes were accompanied by reduced neutrophil infiltration, as reflected by lower MPO activity in both probiotic-treated groups compared with MUC. L156.4 also increased *Muc2* expression, in line with goblet cell preservation, whereas both strains induced only modest, non-restorative changes in tight-junction markers such as *Tjp1*. Consistent with these observations,^55^ and^56^ also reported that lactic acid bacteria-based probiotics attenuate 5-FU induced histological damage and MPO activity.

At the inflammatory level, beyond the reduction of MPO, both strains decreased *Nfkb1*, *Il1b* and *Tgfb1* expression relative to MUC. These effects are consistent with attenuation of NF-κB–driven inflammatory signaling and align with previous reports showing that probiotic lactobacilli can modulate TLR-dependent NF-κB pathways and support epithelial barrier function.^57‐59^ In contrast, *Tnf* remained significantly elevated in MUC animals and was not significantly reduced by either strain, although a trend towards lower values was observed in probiotic-treated mice. Residual TNF activity has been proposed to contribute to epithelial repair rather than simply reflect persistent inflammation,^60,61^ which may help to explain this pattern. *Il10* expression showed a more ambiguous profile: it did not differ between NC and MUC, but was significantly lower in both probiotic groups than in NC and remained comparable to MUC. Given this pattern and the single time point analyzed, it is not possible to determine whether the reduced *Il10* transcription reflects a beneficial rebalancing of the immune response or phase-specific regulation of this cytokine.^62^ showed that chemotherapy-induced dysbiosis can suppress macrophage-derived IL-10 via TLR4–NF-κB and that probiotics may modulate this axis without fully restoring IL-10 levels. In our model, changes in *Il10* are therefore best interpreted as part of a selective immunomodulation, rather than a simple anti-inflammatory effect.

The protective effects of L156.4 and LGG were also reflected at the microbiota level, although in different ways. Mucositis markedly reduced Shannon diversity and Pielou evenness and reshaped community structure, with expansion of *Campylobacterota* and increased *Helicobacter* abundance, consistent with an inflammatory profile.^45,63‐65^ Live LGG largely restored diversity and evenness indices and shifted the overall microbial configuration towards that of NC animals, in line with previous reports of its ability to stabilize gut communities.^66,67^ In contrast, L156.4 did not restore global diversity measures but did significantly reduce *Campylobacterota* abundance, while other genus-level changes were modest and mostly non-significant. Thus, in this model LGG exerted a broader impact on community structure, whereas L156.4 showed a more limited microbiota modulation. Future work should integrate microbiota profiling with measurements of luminal metabolites, such as short-chain fatty acids, to better link these compositional shifts to epithelial protection.

In contrast to the robust effects observed with live preparations, postbiotic fractions of L156.4 and LGG conferred only modest, preparation-specific benefits. Heat-inactivated cells and nonconcentrated cell-free supernatants did not prevent weight loss, intestinal permeability, or substantially reduce histological damage, although some groups showed isolated improvements, such as preservation of intestinal length or reduced MPO activity. These findings are in line with reports of partial or context-dependent protection by postbiotics in mucositis and colitis models.^20,68,69^ A plausible explanation is that nonconcentrated supernatants delivered subtherapeutic levels of bioactive molecules such as SCFAs, peptides, or bacteriocins, consistent with studies showing that concentration or purification is often required to achieve significant activity.^11,70,71^ It is also possible that, in this severe high-dose 5-FU model, postbiotics are less able than live cells to show measurable protection at 72 h. In addition, heat-inactivated cells lack the ability to adhere, sense, and dynamically interact with the epithelium and immune system, or to continuously produce metabolites *in situ*, which are key features of live probiotics^72‐76^ Overall, our data support that, in this severe 5-FU model, live cell activity is critical for meaningful protection.

Despite these constraints, our findings show how two *L. rhamnosus* strains with different origins behave in a stringent chemotherapy-induced mucositis model. Live L156.4 and LGG improved mucosal morphology, reduced neutrophilic inflammation, modulated NF-κB-associated cytokine expression, and partially influenced barrier- and microbiota-related parameters, whereas their postbiotic forms were substantially less effective. The strains exhibited overlapping but distinct functional signatures: LGG more strongly restored global microbial diversity and community structure, whereas L156.4 was associated with a clearer *Muc2*/goblet cell-related profile and specific reductions in proinflammatory taxa such as *Campylobacterota*. These properties underscore the importance of strain-specific evaluation and suggest that optimizing dosing, timing, and formulation for each strain to maximize their complementary therapeutic potential in chemotherapy-induced mucositis.

## Supplementary Material

Supplementary_TableS1S2S3.xlsx Supplementary_TableS1S2S3.xlsx

Supplementary_FigureS1.docx Supplementary_FigureS1.docx

## Data Availability

All raw FASTQ sequencing files have been submitted to the NCBI BioProject repository (accession: [PRJNA1346064]). Count tables and the complete analysis code used for microbiome profiling and statistical analyzes are publicly available at https://doi.org/10.6084/m9.figshare.30375190. Metagenomic and RT-QPCR data are available at https://doi.org/10.57745/2WXOS0 and https://doi.org/10.57745/MBQE4I, respectively.
