# Supplementary material for: Comparative preventive effects of probiotic and postbiotic preparations of Lacticaseibacillus rhamnosus L156.4 and GG in a 5-FU-induced mucositis model
Source: Gut Microbes Rep. 2026 May 21;3(1):2675856. doi: 10.1080/29933935.2026.2675856 (PMC13196637; doi:10.1080/29933935.2026.2675856)
Supplement: Supplementary_FigureS1.docx [file KGMR_A_2675856_SM4266.docx]

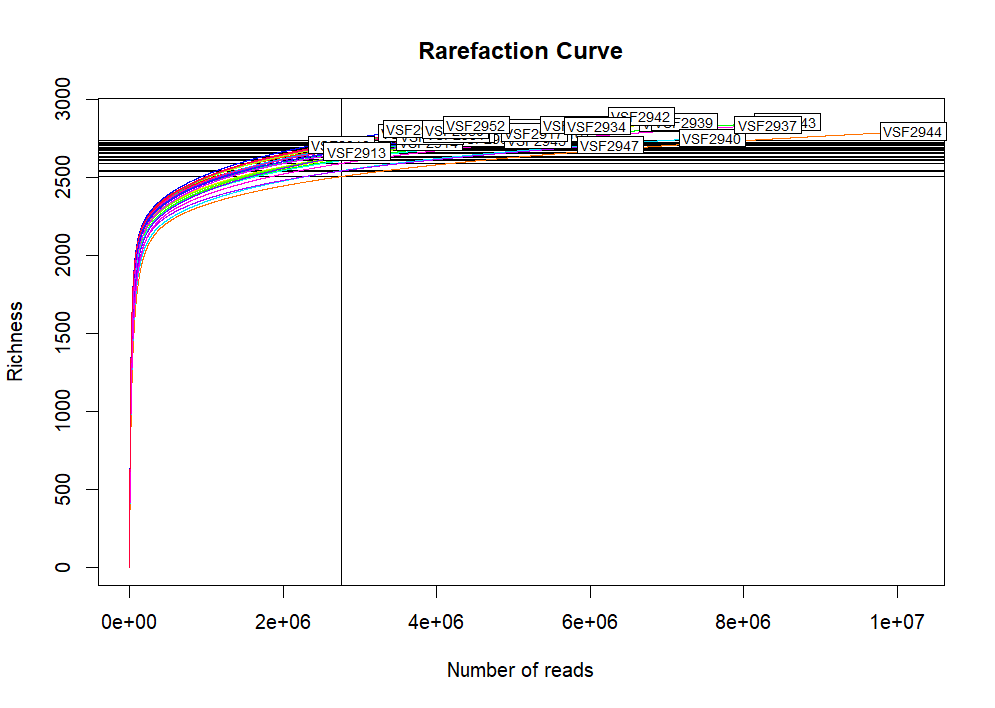


**Figure S1:**  Rarefaction curves showing sequencing depth and species richness of fecal microbiota samples. Each line represents one sample, with the x-axis showing the number of sequencing reads and the y-axis representing observed species richness.
All curves reached a plateau, indicating sufficient sequencing depth and comparable sampling effort among groups. These results confirm that sequencing coverage was adequate for subsequent alpha-diversity analyses.
